# Urgency as a predictor of change in emotion dysregulation in adolescents

**DOI:** 10.3389/fpsyt.2024.1451192

**Published:** 2024-10-03

**Authors:** Lindsey Fisher-Fox, MacKenzie Whitener, Wei Wu, Melissa A. Cyders, Tamika C. B. Zapolski

**Affiliations:** ^1^ Department of Psychology, Indiana University – Indianapolis, Indianapolis, IN, United States; ^2^ Department of Psychiatry, Indiana University School of Medicine, Indianapolis, IN, United States

**Keywords:** negative urgency, positive urgency, emotion dysregulation, adolescents, risk-taking

## Abstract

**Introduction:**

Adolescence is a key developmental period characterized by increased maladaptive risky behaviors. Two related but distinct constructs, urgency (the tendency to act rashly in response to strong negative or positive emotions) and emotion dysregulation, are important risk factors for engaging in maladaptive risky behaviors. Thus far, research has largely agreed that these two risk factors are highly correlated. However, the causal direction between these constructs is less understood. The goal of the current study is to determine whether urgency predicts emotion dysregulation change among adolescents.

**Method:**

This project is an analysis of 544 youth (49.8% female, M_age_=14.22, SD=0.52). We tested whether urgency at baseline predicts change in emotion dysregulation over a nine-week period, and whether that relationship differs across boys and girls.

**Results:**

Two multigroup latent change score path analyses found that negative, but not positive, urgency significantly predicted emotion dysregulation change (negative urgency: *b*= -0.57, *p*=0.001; positive urgency: *b*=0.22, *p*=0.06). There was no evidence of moderation by gender.

**Discussion:**

This work provides initial evidence of a temporal relationship between higher negative urgency and increased emotion dysregulation. The next step is to determine whether negative urgency imparts risk for maladaptive behaviors through its effect on emotion dysregulation. The long-term goal of this program of research is to design and test interventions to reduce the impact of negative urgency for adolescent risk-taking.

## 1 Introduction

Adolescence is a key developmental period for the emergence and development of maladaptive risky behaviors (1–4) and is associated with increased emotional lability and risk-taking behaviors (5). Adolescents show marked increases in drinking alcohol, drinking and driving, using substances, engaging in risky sexual behaviors, and many other risk behaviors (2, 6–10). Two related but distinct constructs, urgency (the tendency to act rashly in response to strong negative or positive emotions) and emotion dysregulation, are important risk factors for engaging in maladaptive risky behaviors (11–13). Thus far, research has largely agreed that these two risk factors are related (14–17); however, the causal direction between these constructs is less understood. The goal of the current study is to determine whether urgency predicts emotion dysregulation change among adolescents.

Negative and positive urgency (18) reflect individual difference tendencies toward rash or maladaptive action during extreme emotional states. In adolescents, negative urgency relates to a wide range of maladaptive risk taking, including suicide attempts and non-suicidal self-injury, as well as the onset of binge eating and alcohol, marijuana, cigarette, and other drug use (19–29). Similarly, positive urgency relates to non-suicidal self-injury, and predicts the onset of cigarette, marijuana, and alcohol use (20, 22, 25, 28, 29). Despite these well-established relationships, the mechanism(s) by which negative and positive urgency impart risk are less well understood.

Emotion dysregulation, defined as engaging in a behavior to cope with emotions that is producing a dysfunctional, rather than an adaptive, outcome (30), may be a prime mechanism for how urgency increases maladaptive risk taking in adolescents. Emotion dysregulation is a hallmark symptom of and risk factor for psychopathology (13, 31–33). In youth, emotion dysregulation predicts aggressive behavior, deliberate self-harm, risky sexual behaviors, substance use, and eating pathology (13, 34–40). Interestingly, one study found that psychopathology does not, in turn, predict increases in emotion dysregulation (13), suggesting that emotion dysregulation may be a precursor of psychopathology and not the other way around.

Research has established that urgency and emotion dysregulation are related constructs, with moderate to strong bivariate correlations (r=0.32-0.70) (14–17, 41–47). Some work has conceptualized urgency as poor emotion regulation (48), with others supporting relationships between urgency and the use of fewer appropriate, and more inappropriate, emotion regulation strategies (49). Research has found that negative and positive urgency are significantly associated with emotion dysregulation (16), but that emotion dysregulation is not significantly associated with urgency (15). Importantly, these studies utilized cross-sectional data, so the temporal order of the relationship between urgency and emotion dysregulation cannot be inferred, leaving the causal direction between these constructs unknown.

Additionally, gender may impact the relationship between urgency and emotion dysregulation. First, boys more likely to engage in risky behavior than girls (50). Second, girls begin using emotion regulation strategies more quickly (51) and experience more emotion dysregulation (52, 53) and less emotional clarity, whereas boys have more difficulty with emotional awareness (52, 53). Third, there is some evidence that males may have higher levels of positive urgency (54), and females may have higher levels of negative urgency (55, 56), although some studies have failed to find gender differences in urgency (57, 58).

## The current study

The goal of the current study is to determine whether negative and positive urgency predict emotion dysregulation change in adolescents. The underlying theoretical model for the current study proposes that trait urgency is an underlying predisposition that leads to the development of maladaptive risky behaviors, while emotion dysregulation is a set of skills (or lack thereof) that develops in part due to the underlying urgency predisposition, which then further reinforces maladaptive risk. Alternative conceptualizations exist, including conceptualizing urgency and emotion dysregulation as one and the same (14, 15, 48, 59–61). In the absence of experimental or longitudinal work establishing this pathway direction, we relied on theory (18) suggesting urgency as the precursor, as well as general evidence that personality develops temporally earlier (62–65) than emotion dysregulation (51, 66, 67).

## Hypotheses

The hypotheses for the current study, supported by the reviewed literature, are as follows:

Hypothesis 1: Baseline negative urgency will significantly predict change in emotion dysregulation from baseline to the follow-up, such that negative urgency will be associated with increased emotion dysregulation. Gender will moderate the relationship, such that the relationship will be stronger in girls than in boys.

Hypothesis 2: Baseline positive urgency will significantly predict change in emotion dysregulation from baseline to the follow-up, such that positive urgency will be associated with increased emotion dysregulation. Gender will moderate the relationship, such that the relationship will be stronger in in girls than in boys.

## Methods

This study is a secondary analysis of the Going 4 Goals project (PI: Zapolski), which seeks to determine the effectiveness of a brief adaptation of the skills group component of dialectical behavioral therapy for adolescents (DBT-A) to reduce risky behaviors among high school students who were engaging or at risk of engaging in high-risk behaviors, such as substance use, as identified by school staff (see protocol for full description of Going 4 Goals project, 68). The Going 4 Goals Project included a control sample of students who did not participate in the program but were included to compare changes in study outcomes to those students who did participate in the program. This study utilizes only the control sample from the parent study who did not receive any DBT-A skills training to eliminate systematic differences between the control and intervention groups due to the DBT-A intervention or pre-morbid risk profiles.

## Participants

Participants were 544 ninth-grade high school students who ranged in age from 13 to 15 (49.8% female, M_Age_=14.22, SD=0.52) from a local public high school in Indianapolis recruited during the school’s health class at the beginning of either the Fall or Spring semester (between Fall of 2018 and Spring of 2020). Participants were offered the opportunity to participate while enrolled in a state-mandated health education class at their school.

## Measures

### Emotion dysregulation

The Emotion Dysregulation Scale short version (EDS-s) is a 12-item self-report measure that examines the construct of emotion dysregulation across three domains: emotional experience, cognition, and behavior (69). Items were scored on a 7-point Likert scale ranging from 1 (not true) to 7 (very true). Example items include “emotions overwhelm me” and “when I’m upset, I have trouble seeing or remembering anything good about myself.” This scale was found to have high internal consistency in the current sample (Cronbach’s alpha = 0.96), which is consistent with previous research [Cronbach’s alpha = 0.93 – 0.95; (69)]. This total score was calculated using a sum of the items. The EDS was completed at baseline and nine weeks later.

### Negative urgency

Negative urgency was measured using the negative urgency subscale of the full UPPS-P modified for children [UPPS-PC; (70)]. Items were measured on a 4-point Likert scale from 1 (not at all like me) to 4 (very much like me), such that higher scores are indicative of more impulsive tendencies. One example item is, “When I feel bad, I often act without thinking.” The UPPS-PC uses eight items to assess negative urgency. This subscale was found to have good internal consistency in the current sample (Cronbach’s alpha = 0.85), which is consistent with previous research of the full UPPS-PC [Cronbach’s alpha = 0.81-0.90; (70)]. The total negative urgency score was calculated using a sum of items. Data from the baseline session was used for data analysis.

### Positive urgency

Positive urgency, a component of impulsivity, was measured using the positive urgency subscale of the full UPPS-PC (UPPS-PC; 70). The items were measured on a 4-point Likert scale from 1 (not at all like me) to 4 (very much like me), such that higher scores are indicative of more impulsive tendencies with items such as “when I get really happy about something, I tend to do things that lead to trouble.” The UPPS-PC uses eight items to assess positive urgency. This subscale was found to have high internal consistency in the current sample (Cronbach’s alpha = 0.9), which is consistent with previous research with the UPPS-PC (Cronbach’s alpha = 0.81-0.90; 70). The total positive urgency score was calculated using a sum of items. Data from the baseline session was used for data analysis.

## Procedure

The school staff sent all students an opt-out consent form letter for parent and/or guardian approval. The letter contained the study purpose, risks, benefits, and inclusion and exclusion criteria. Guardians were asked to sign and return the letter if they did not wish their student to participate and were given a period of two weeks to return the letter. After the two-week period passed, students who wished to participate signed assent forms and completed surveys assessing baseline measurements of the outcome variables. These measures were then collected again nine weeks later. Researchers provided snacks to study participants as incentives for completing the baseline and follow-up surveys.

## Data analysis

All analyses were performed in R (71).

### Data cleaning

Before beginning data analysis, participants were removed due to missing data at baseline, not providing a response to the gender item, or not identifying as cisgender male or female: Six participants were removed from the data set for not providing a response to the gender demographics item, and an additional five participants were removed for responding as transgender or “other” gender. While it is important to examine gender beyond the male and female binary, the sample of other genders was not large enough to be adequately powered to determine an effect; thus, the decision was to remove them for this analysis. There were 5 other participants removed from the data set due to missing the EDS-s or UPPS-PC at baseline. After removing those participants, there were 528 remaining participants for analysis.

### Missing data analysis

The data set was then assessed for missingness. A test for missing completely at random (MCAR) was conducted on each of the variables of interest using Little’s test statistic (72). They were each non-significant, indicating that the data missing in the EDS-s and the UPPS-PC were MCAR. The EDS-s was found to have 0.4% missing data at baseline and 12% missing data at follow-up. The negative urgency subscale and positive urgency subscale each had 0.3% missing data at baseline. Upon a visual inspection of the data set, the missing data at the baseline time points were for individual items within the measures. Thus, given the small amount of missing data and MCAR mechanism, we calculated scale scores using the person mean imputation approach (73). Specifically, a total scale score was calculated for the EDS-s at baseline and follow-up, negative urgency at baseline, and positive urgency at baseline by taking a sum of the individual item scores for each participant. Note that those scale scores were only used in descriptive and preliminary analyses. For the confirmatory factor and latent change score models described below, the target constructs were included as latent variables with missing data on the individual items handled using the full information maximum likelihood estimation method (FIML).

### Preliminary analyses

All variables were assessed for normal distribution skewness, kurtosis, and outliers. Previous research indicates that skewness between -2 and +2 and kurtosis between -7 and +7 are considered to be within a normal distribution range (74, 75). Bivariate correlations, t-tests, and an ANOVA were conducted to examine the associations between positive urgency, negative urgency, emotion dysregulation, and sample characteristics with the “psych” package in R (76).

### Measurement invariance

Confirmatory factory analysis (CFA) was used to assess the factor structure of the EDS-s at baseline and follow-up and the negative and positive urgency items of the UPPS-PC using the “lavaan” package in R (77). A single-factor model was first fit to the data for each construct with the gender groups combined. Each latent construct was identified using the indicator approach (i.e., fix one of the item loadings to be 1). Model fit was assessed using the comparative fit index (CFI) and the root mean squared error or approximation (RMSEA): RMSEA<0.08 and CFI >0.90 were deemed adequate (78, 79). To ensure that the constructs were comparable across gender groups, measurement invariance of each latent construct between boys and girls was then evaluated using a series of multigroup CFA analyses (78) by sequentially adding equality constraints on parameters across groups: 1) configural (no parameter constraints), 2) weak invariance (factor loadings equated), and 3) strong invariance (factor loadings and intercepts equated). Configural invariance was established if the model fit the data. Weak and strong invariance were evaluated using chi-squared difference tests (Δχ^2^), as well as changes in CFI and RMSEA (ΔCFI and ΔRMSEA). While there are no set cut-off criteria, the current standard is to accept models that show ΔCFI and ΔRMSEA ≤.01 (80). Note that for the purpose of the current study (compare relationships among latent variables across groups), weak invariance would be sufficient.

Invariance of the EDS-s across time (baseline to follow-up assessments) was evaluated using the same process of adding equality constraints across the two assessment periods (81): 1) configural (no parameter constraints), 2) weak invariance (factor loadings equated), and 3) strong invariance (factor loadings and intercepts equated). The same fit indices as previously mentioned were used to evaluate model fit.

### Hypothesis testing

**Figure 1:**
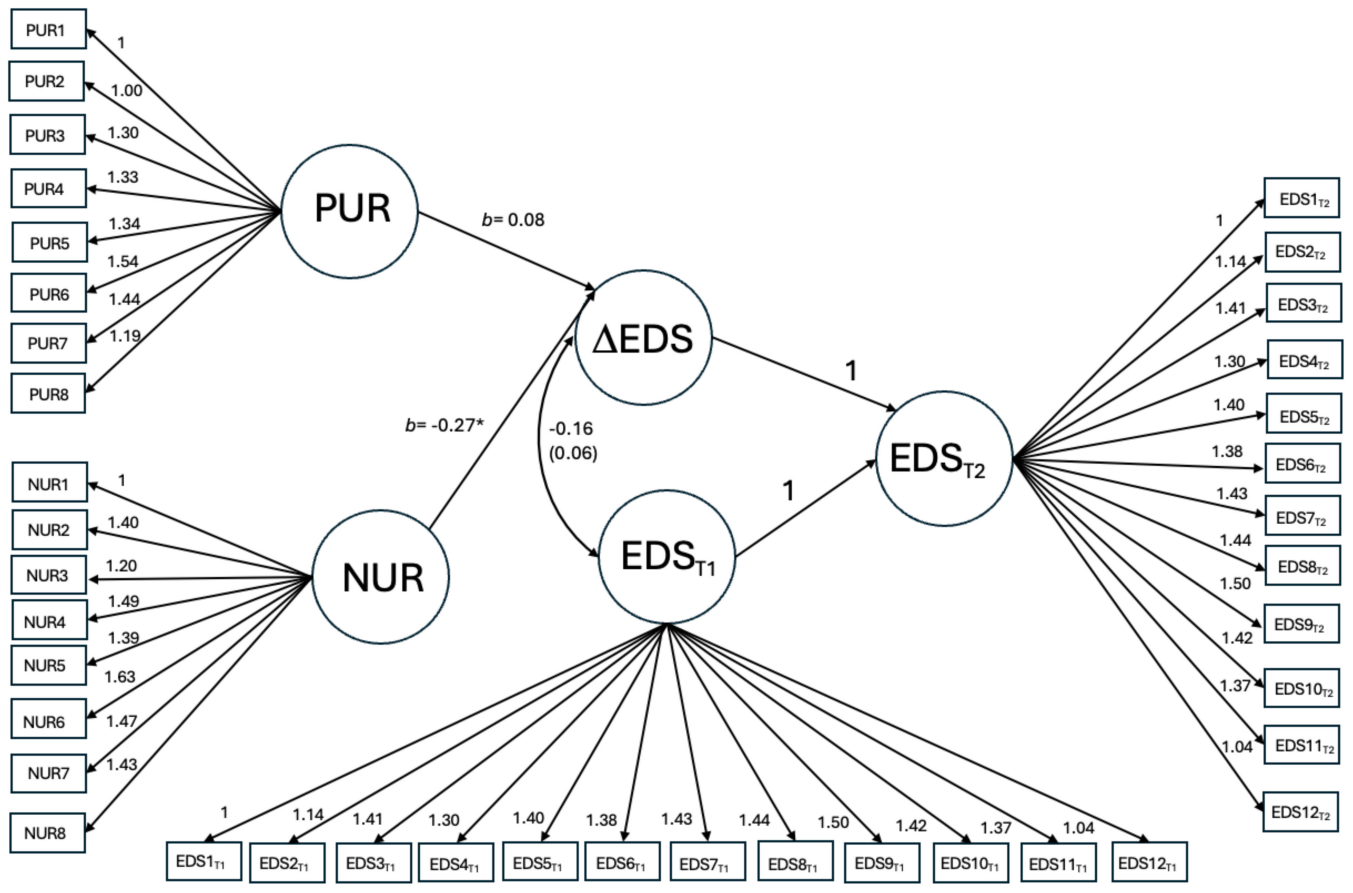
Path diagram for the multiple group latent change score model with regression coefficients associated with negative and positive urgency freely estimated across gender. For emotion dysregulation, since it was measured at two time points, the residuals on the same item were allowed to covary across time. For simplicity, these covariances were included in the model but omitted from the graph. Results were similar comparing unconstrained to models with only negative urgency or only positive urgency constrained, although they trended towards having worse fit. NUR=negative urgency, PUR=positive urgency, EDS_T1_= EDS-s at baseline, EDS_T2_= EDS-s at follow-up, ΔEDS = change in EDS-s from baseline to follow-up. *p<0.05.

After establishing weak invariance held for all the constructs, two multigroup latent change score models (see **Figure 1** for the model specifications) were built to test whether baseline negative and positive urgency predicted change in emotion dysregulation from baseline to follow-up and if the associations differed by gender. All models were estimated using FIML for missing data with the “lavaan” package in R (77). The latent change score models modeled the change as a latent variable, removing the influence of measurement errors and facilitating the use of FIML in dealing with missing data on difference scores (82, 83). The first model allowed the associations to be different across boys and girls, and the second one equated them across groups. Note that for emotion dysregulation, since it was measured at two time points, the residuals on the same item were allowed to be correlated across time. The two models were nested and compared using Δχ^2^, with a significant result suggesting a moderation effect of gender (i.e., the associations significantly differed across group). As a sensitivity analysis, an additional equality constraint model was run on both positive and negative urgency, in separate models, such that the coefficients were constrained to be equal for negative, but not positive urgency, and vice versa, allowing to examine whether gender moderation occurred for one trait, but not the other. Cohen’s guidelines for coefficient *β* were used to determine the effect size of the relationships between negative and positive urgency and emotion dysregulation (84).

## Results

### Sample characteristics

Participants were between the ages of 13-16 and, on average, 14 years old (M=14.21, SD=0.52); 74% of the participants were 14 years old. Because of this, age was not included as a covariate in hypothesis testing. The sample was mostly Black or White (31.3% African American/Black, 27.4% White, 0.4% Asian American/Pacific Islander, 1.9% Native American/American Indian/Alaskan Native, 6.3% Other race, and 18.7% more than one race, 24.2% did not respond to race demographic item), mostly not Hispanic/Latino (25.1% Hispanic/Latino), and in ninth grade (100%).

### Preliminary analyses

Skewness and kurtosis were within normal limits for each study variable. Means, standard deviations, and correlations of study variables can be found in **Table 1**. As expected, negative urgency, positive urgency, and emotion dysregulation (both baseline and follow-up), showed positive, significant correlations (all *p*’s<0.001). Boys demonstrated lower negative urgency [*t*(526)=-3.23, *p*=0.001; boys: M=18.21, SD=5.13 and girls: M=19.76, SD=5.82)] and emotion dysregulation at both baseline [*t*(526)= -6.39, *p*<0.001; boys: M_1_ = 36.72, SD_1_ = 16.41; girls: M_1_ = 46.55, SD_1_ = 18.88] and follow-up [*t*(458)= -6.02, *p*<0.001; boys: M_2_ = 35.92, SD_2_ = 17.26; girls M_2_ = 46.06, SD_2_ = 18.84) than girls. There was no difference in positive urgency across gender [*t*(526)= -0.20, *p*=0.84].

**Table 1:** Means, standard deviations, and correlations of key study variables.

Variable	1	2	3	4	5
1. Age	–				
2. NUR	0.04	–			
3. PUR	0.03	0.55***	–		
4. EDS baseline	0.02	0.66***	0.37***	–	
5. EDS follow-up	0.02	0.57***	0.33***	0.79***	
Mean (SD)	14.22 (0.52)	18.98 (5.54)	17.04 (5.81)	41.62 (18.34)	40.99 (18.75)

NUR, negative urgency; PUR, positive urgency; EDS, emotion dysregulation scale-short.

***p<0.001.

### Measurement invariance

First, the factor structure for the EDS-s at baseline and at follow-up supported a single factor fit the data with the two groups combined (baseline: RMSEA = 0.073, 90% CI = [0.063, 0.084], CFI = 0.96; follow-up: RMSEA = 0.058, 90% CI [0.046, 0.070], CFI = 0.98). Three residual covariances were included across six items (items five and nine, items 11 and 12, and items one and nine). For negative negative urgency, two residual covariances were included (items 17 and 26 and items 20 and 30) and a single factor fit the data with the two groups combined (RMSEA = 0.067, 90% CI [0.049, 0.086], CFI = 0.97). For positive urgency, six residual covariances were included (items 38 and 39, items 36 and 40, items 33 and 34, items 35 and 38, items 34 and 36, and items 33 and 36) and a single factor fit the data with the two groups combined (RMSEA = 0.080, 90% CI [0.060, 0.101], CFI = 0.98).

Second, measurement invariance analyses were conducted for each construct (see **Table 2**). The configural invariance models with the multiple group CFA also showed an adequate fit. The models with factor loadings equated did not have a significantly different model fit from the corresponding configural models, supporting weak invariance. The strong invariance models showed significantly worse χ^2^ for all constructs except for positive urgency. Although ΔCFIs from weak to strong invariance were less than.01 for most of the constructs, ΔRMSEAs were all greater than the threshold, indicating that strong invariance was not established. Based on the result, weak invariance was assumed. Correspondingly, in subsequent hypothesis testing models, item loadings were constrained to be equal across boys and girls, but intercepts were not.

**Table 2:** Measurement Invariance for Sex (dummy coded against Male).

	CFI	RMSEA [90% CI]	χ^2^	ΔCFI	ΔRMSEA	Δχ^2^
EDS-s Baseline						
Configural	0.96	0.074 [0.063, 0.086]	250.005**			
Weak	0.96	0.070 [0.059, 0.081]	259.357**	0.000	0.000	9.863
Strong	0.96	0.070 [0.059, 0.081]	283.992**	0.004	0.069	24.635*
EDS-s Follow-up						
Configural	0.97	0.065 [0.051, 0.078]	200.587**			
Weak	0.97	0.061 [0.048, 0.074]	209.683**	0.001	0.000	9.096
Strong	0.97	0.063 [0.051, 0.075]	237.149**	0.007	0.081	27.466**
EDS-s Across Time						
Configural	0.93	0.070 [0.065, 0.075]	870.190**			
Weak	0.93	0.068 [0.063, 0.073]	873.662**	0.001	0.000	3.472
Strong	0.93	0.066 [0.061, 0.071]	883.858**	0.000	0.000	10.195
Negative Urgency						
Configural	0.97	0.067 [0.046, 0.087]	78.281**			
Weak	0.97	0.060 [0.040, 0.079]	83.445**	0.002	0.000	5.164
Strong	0.95	0.068 [0.051, 0.085]	111.726**	0.016	0.107	28.281**
Positive Urgency						
Configural	0.98	0.085 [0.063, 0.106]	80.805**			
Weak	0.98	0.072 [0.052, 0.092]	82.365**	0.002	0.000	1.560
Strong	0.98	0.068 [0.049, 0.087]	93.272**	0.001	0.046	10.907

*p<0.05, **p<0.01.

Third, configural invariance of the EDS-s across time showed an adequate fit (see **Table 2**). The model with the factor loadings equated did not have a significantly different model fit from the corresponding configural model, supporting weak invariance. The strong invariance model did not have a significantly different model fit from the configural model, supporting strong invariance. Thus, strong invariance was established for the EDS-s across time, allowing to the examination of change over time.

### Hypothesis testing

The multigroup latent change score analyses found no significant moderation effect of gender. Between the models with the regression coefficients associated with negative and positive urgency equated *vs*. relaxed across boys and girls, there was no significant change in model fit (Δχ^2^ = 3.785, Δdf = 2, *p*=0.15). The constrained model (RMSEA = 0.062, 90% CI [0.059, 0.065], CFI = 0.89) revealed that negative urgency (*b*= -0.27, *p*=0.02) was significantly associated with change in emotion dysregulation, while the relationship with positive urgency was not significant *(b*=0.22, *p*=0.06; see **Figure 1**). There was also no significant change in model fit when only negative or positive urgency had their coefficients constrained to be equal, although it did trend towards significance (positive urgency coefficients constrained model to unconstrained model: Δχ^2^ = 3.219, Δdf = 1, *p*=0.07; negative urgency coefficients constrained model to unconstrained model: Δχ^2^ = 3.070, Δdf = 1, *p*=0.08).

## Discussion

This study was the first to examine a temporal, predictive relationship between urgency and emotion dysregulation change. Results indicated that baseline negative, but not positive, urgency predicted change in emotion dysregulation across a 9-week period among our sample of 9^th^ grade adolescents. Girls had higher emotion dysregulation scores than boys at both timepoints; however, there was not a main effect of gender in either model, and gender did not significantly moderate the relationship between urgency and emotion dysregulation change.

The negative, significant relationship between baseline negative urgency and change in emotion dysregulation extends previous cross-sectional relationships between negative urgency and emotion dysregulation (14, 16, 17, 42, 44, 46), suggesting that higher negative urgency is associated with increased emotion dysregulation over time. This study provides initial evidence of a temporal relationship, supporting the idea that negative urgency influences the development of emotion dysregulation over time, as suggested by previous theory (18, 64, 65). If this is true, negative urgency may impact how one learns to regulate their emotions through personality-environment translation effects and may serve as one mechanism for how negative urgency impacts risk [e.g., (18)].

The relationship between baseline positive urgency and change in emotion dysregulation was not significant, which is consistent with previous research that indicates that positive and negative urgency relate to some risky behaviors in different ways (15, 47, 85–87). This finding contradicts previous work that suggests that both negative and positive urgency relate to emotion dysregulation (e.g., 42, 44) and have similar risk patterns (11, 88, 89). The effect of positive urgency on emotion dysregulation change fell just short of significance, and was in the opposite direction than hypothesized and than the effect of negative urgency. This may mean that positive urgency has a unique, and as of yet unstudied and not understood, negative impact on emotion dysregulation. However, given the trend-like nature of this effect and the unexpected direction, this should be examined more fully before reaching this conclusion. The use of the EDS-s, which primarily includes items concerning negative emotional states, may have contributed to significant association of negative urgency and null effects of positive urgency in the current study. Future research regarding the relationship between emotion dysregulation and positive urgency may consider assessing emotion dysregulation with positive emotions, such as the DERS-positive (90). Alternatively, since both negative and positive urgency were placed into one model, the residual variance trend with positive urgency, after removing the effect of negative urgency, may be spurious, especially given the high intercorrelation between the two traits.

Although negative urgency had a significant association on emotion dysregulation change, the effect size was small (84) and the study period was brief. Although statistically significant, the small effect may not translate to clinically-significant effects. On the other hand, small effects can be important when they occur with minimal manipulation or when they impact a difficult-to-change outcome (91). Thus, although the effect in this study was small, the fact that it occurred over such a short period of time and without intervention suggests that this effect could be meaningful in broader prospective or interventional studies. A longer follow-up period could provide additional insight into the true impact of urgency on emotion dysregulation across the adolescent period. First, emotion dysregulation develops in early and middle adolescence (51, 52, 67), but the time over which measurable, natural change in emotion dysregulation takes place is less well understood. The brief nine-week period used in the current study may have resulted in smaller effects as there was less change in emotion dysregulation to predict. Second, we chose the direction from urgency to emotion dysregulation based on theory that personality develops temporally before emotion dysregulation, which suggests that urgency may be the precursor (51, 62–67). However, this does not rule out a feedback loop from emotion dysregulation back to changes in urgency, which could then further impact changes in emotion dysregulation over time. Alternatively, others conceptualize negative urgency as being part of emotion dysregulation (14, 48, 61). These additional models should be studied further in future work.

Girls had higher emotion dysregulation scores compared to boys. This finding contradicts prior work that suggests there are no gender differences in emotion dysregulation (92, 93), but supports other findings that girls experience more emotion dysregulation compared to boys (52, 53). This is thought to be due to adolescent boys being less aware of their emotional experience than girls (52, 53). There is extensive work establishing that boys have elevated levels of risk-taking compared to girls (50). For example, adolescent boys are more likely to use drugs (94) and to gamble (95) than adolescent girls. These higher levels of negative urgency and emotion dysregulation in girls found in the present study could lead to higher risk for girls in other domains, such as anxiety and depression (49, 96).

Findings did not support the hypothesis that gender would moderate the relationship between urgency and emotion dysregulation, suggesting that negative urgency relates to emotion dysregulation in the same way across boys and girls. It is unlikely that the null result in the current study was driven by statistical concerns because the large sample allowed for adequate power to test for a small effect and because there was an equal proportion of boys and girls in the sample. However, and importantly, there was a significant relationship between negative urgency and gender, which could mask interaction effects.

The long-term goal of this line of research is to determine how to reduce risk-taking in adolescents. Given the high rate of risk-taking and emotional lability among adolescents (5), this goal addresses an important intervention endpoint. Negative and positive urgency may not be directly intervenable and may even impede treatment response (97–100). Emotion dysregulation could be targeted as a modifiable risk factor (45) to reduce the impact of negative urgency on emotion dysregulation development. One study has sought to do this: Weiss et al. (17) found success in reducing both emotion dysregulation and urgency through an emotion modulation skills training. This study, along with the current findings, suggest that future research should test whether or not existing effective emotion regulation treatments [see (101)] can also successfully reduce urgency and its impact of risk-taking. A more immediate and practical application of the current study is that clinicians may want to measure negative urgency and emotion dysregulation constructs in youth to better understand why, and under what conditions, youth engage in risk-taking. Both the UPPS-PC and the EDS-s appear to be adequate measures of these constructs in youth that are freely available and require very little time to implement, thus maximizing the benefits and minimizing the costs of additional assessments.

This study is not without limitations. First, we relied on self-report measures, which are limited by how aware, open, and willing participants were to disclose. Emotional awareness is less developed in adolescence (52), which could lead to under-reporting of both urgency and emotion dysregulation. Under-reporting could have reduced the effect size between these constructs detected in the current study. Using caregiver and/or teacher reports and behavioral measures, such as respiratory sinus arrhythmia (33), would provide complementary and potentially more robust relationship effects. Second, this sample was primarily composed of cisgender youth, which may limit generalizability to other gender identities. Third, strong measurement invariance was not fully supported for negative urgency and the EDS-s, suggesting that although comparisons can be made comparing strengths of relationships across boys and girls, boys and girls show mean level differences in these traits and comparisons should only be made with this in mind. Fourth, there could be a third variable responsible for the changes seen in emotion dysregulation across time, such as neuroticism or negative affectivity, which was not examined in the current study. One study found that neuroticism is typically higher in adolescent females than males starting around age 14 (102). Fifth, although participants in this study likely had a wide range of risk behavior engagement, this work should be replicated in clinical samples to ensure generalizability to high-risk adolescents.

In conclusion, this study is the first to establish a predictive temporal relationship between negative urgency and increased emotion dysregulation in adolescents, albeit in a brief timeframe. This work extends previous cross-sectional research and suggests the viability of further prospective work examining this relationship over a longer period of time, incorporating a measurement of risk-taking as the endpoint outcome. Positive urgency may relate to emotion dysregulation differently and should be studied further. Understanding how these constructs are related, and in turn, relate to the development of risky behaviors in adolescents, paves the way for the design and testing of interventions to reduce the impact of negative urgency for adolescent risk-taking.

## Data Availability

The data analyzed in this study is subject to the following licenses/restrictions: Identifiable data on participants under the age of 18. Requests to access these datasets should be directed to Tamika Zapolski, tzapolsk@iu.edu.
